# Variable ambient temperature promotes song learning and production in zebra finches

**DOI:** 10.1093/beheco/arad014

**Published:** 2023-03-19

**Authors:** Maëlle Lefeuvre, ChuChu Lu, Carlos A Botero, Joanna Rutkowska

**Affiliations:** Jagiellonian University, Faculty of Biology, Institute of Environmental Sciences, Cracow, Poland; Jagiellonian University, Doctoral School of Exact and Natural Sciences, Cracow, Poland; Jagiellonian University, Faculty of Biology, Institute of Environmental Sciences, Cracow, Poland; Jagiellonian University, Doctoral School of Exact and Natural Sciences, Cracow, Poland; University of Texas at Austin, Department of Integrative Biology, Austin, TX, USA; Jagiellonian University, Faculty of Biology, Institute of Environmental Sciences, Cracow, Poland

**Keywords:** condition mismatch, parental effects, song learning, temperature, variability

## Abstract

Current climate change is leading to increasingly unpredictable environmental conditions and is imposing new challenges to wildlife. For example, ambient conditions fluctuating during critical developmental periods could potentially impair the development of cognitive systems and may therefore have a long-term influence on an individual’s life. We studied the impact of temperature variability on zebra finch cognition, focusing on song learning and song quality (*N* = 76 males). We used a 2 × 2 factorial experiment with two temperature conditions (stable and variable). Half of the juveniles were cross-fostered at hatching to create a mismatch between pre- and posthatching conditions, the latter matching this species’ critical period for song learning. We found that temperature variability did not affect repertoire size, syllable consistency, or the proportion of syllables copied from a tutor. However, birds that experienced variable temperatures in their posthatching environment were more likely to sing during recordings. In addition, birds that experienced variable prenatal conditions had higher learning accuracy than birds in stable prenatal environments. These findings are the first documented evidence that variable ambient temperatures can influence song learning in zebra finches. Moreover, they indicate that temperature variability can act as a form of environmental enrichment with net positive effects on cognition.

## Teaser

Climate change and environmental unpredictability can affect cognitive development and subsequent cognitive performance in animals. This effect could be even more severe when learning of a crucial skill occurs within a definite period of time during development. In this study, we show that temperature variability actually enhances song learning accuracy in zebra finches and does not impact other key song parameters, such as the repertoire size or the proportion of syllables copied from the tutor.

## INTRODUCTION

Current climate change is characterized by increasingly unpredictable extreme events, including hot and cold waves, hurricanes, storms, and tsunamis, which imposes an intense pressure on wildlife (Coumou and Rahmstorf 2012; Cohen et al. 2021). Consequences of those events, such as modifications of abiotic conditions, food sources, or species interactions, can affect animals directly and indirectly through their survival, development, reproduction, and health (Popic and Wardle 2006; Raffel et al. 2013; Dueñas et al. 2021, see Acevedo-Whitehouse and Duffus 2009; Milligan et al. 2009; Wardle et al. 2013; Sattar et al. 2021 for reviews). For instance, unpredictable weather leads to an unstable invertebrate resource for shorebird chicks over the years and negatively impacts their survival rate (Saalfeld et al. 2019). Maladaptive behaviors in response to those changes can result in lower individual fitness and higher extinction risk (e.g., Glądalski et al. 2018). Conversely, adaptive behavioral and physiological responses to extreme conditions, such as migration or shifts in developmental stages, may ensure population persistence even under suboptimal conditions (e.g., Munn et al. 2010; Couriot et al. 2018).

Parental effects, the ability of mothers and fathers to influence the phenotype of their offspring, are important mechanisms of adaptation. Although maternal effects have received more attention, both parents can prepare their progeny to the environment they will probably face, through gamete quality, provisioning of resources, and pre- and postnatal breeding behaviors (see Mousseau and Fox 1998; Monaghan 2008; Griffith and Buchanan 2010; Groothuis and Taborsky 2015; Rutkowska et al. 2020 for reviews). Thereby, parents are able to signal current environmental conditions to their offspring from the first stages of development, during pregnancy or egg production. Environmental factors encountered by the mothers influence the developmental rate (reviewed in van der Waaij et al. 2011; Giordano et al. 2014; Ruuskanen et al. 2016), reproductive success (Forstmeier et al. 2004; Naguib et al. 2006), morphology (Groothuis et al. 2005), physiology (Nord and Giroud 2020), and behavior of their progeny (Weinstock 2008; Schoech et al. 2011). Yet, due to the time gap between conception and birth, the environmental conditions that offspring experience after birth may not correspond to the ones their parents prepared them for (Lindström 1999). This mismatch has consequences on development and fitness. For instance, food deprivation of mothers leads to a compensatory growth and to intensified fat storage of their juveniles when those are provided with ad libitum food, compared with juveniles from nonrestricted mothers (Remacle et al. 2011; van der Waaij et al. 2011). In fact, being prepared for a certain environment can be quite costly if the actual conditions experienced later are not what was originally anticipated (Costantini et al. 2014).

Maternal effects have also been known to shape learning abilities in the offspring, an important aspect of cognition (e.g., Eaton et al. 2015; Colson et al. 2019). For instance, both pre- and postnatal maternal effects influence spatial learning and memory of mouse pups (reviewed in McCarty 2017). Similarly, testosterone levels in bird eggs can impact embryo auditory learning (Bertin et al. 2009). However, to our knowledge, the relationship between maternal effects and posthatching learning abilities in birds has not yet been described.

Song is a crucial feature for many bird species. This cognitive trait is the result of a learning process that can occur, depending on the species, throughout life or only during a short and close-ended period of time during development. A close-ended learning period implies that disrupting conditions during this period could have irreversible consequences on the song and attractiveness of an individual (reviewed in Riebel 2009). Zebra finch (*Taeniopygia guttata*), a model bird in the field of song learning studies, belongs to the second category. Only males sing in this species, and the main goal of zebra finch song is to attract a mate (Riebel 2009). Most studies on zebra finch song have investigated directed song (i.e., the song produced towards a female during courtship), which differs from undirected song by its higher number of repetitions of introductory notes and motifs, and its higher consistency and song rate (see Riebel 2009 for a review). Zebra finch chicks learn a sequence of song syllables from a tutor, which is most of the time the male that raises them (Mann and Slater 1995; Roper and Zann 2006), during a critical period occurring between 30 and 65 days posthatching. This phase ends with a dramatic decrease of neural plasticity leading to a sensorimotor phase in which juveniles practice and elaborates their own song based on the template they have memorized. At independence, song is crystallized and considered as definitive (see London 2019 for a review).

Temperature has been suggested to influence song production at adulthood; exposure to high temperature shortens song and decreases its consistency in zebra finches (Coomes and Derryberry 2021). Yet, although many species, including zebra finches, experience a high variability of ambient temperature in the wild (e.g., Mariette and Buchanan 2016), the relationship between temperature variability and song learning by juveniles has not been directly studied. It can be expected that temperature variation increases thermoregulation which may be prioritized over brain development and learning (reviewed in Roth et al. 2010). Indeed, in the temperature range of 10–28 °C, zebra finch metabolic rate differs approximately 2-fold (Vleck 1981) and sudden changes in ambient temperature increase mortality especially in the young age classes (Briga and Verhulst 2015). It is unknown how mothers (and fathers) can buffer the consequences of this developmental stress and influence the song learning of their offspring. So far, little work drawing a relationship between parental effects and song learning abilities in birds has been published. Only a few mechanisms were investigated, with opposite results: the egg laying order (Tchernichovski and Nottebohm 1998), but not the hatching asynchrony (Campbell et al. 2017) has been shown to impact the song learning accuracy in zebra finches. Egg androgen and testosterone concentrations have also been considered, with no effect on song quality (Müller et al. 2008; Müller and Eens 2009). In song sparrows, parental conditions affected the song complexity of the offspring, with sons of food-supplemented parents having a smaller repertoire size than sons of nonsupplemented parents (Zanette et al. 2009).

In this study, we addressed the question of whether temperature variability (stable vs. variable conditions) affects song learning and quality in zebra finches. We hypothesized that variable ambient conditions during development can lead to cognitive impairment in song learning, possibly through a prioritized allocation of resources to direct survival rather than brain development and learning capacities. We predicted that juveniles developing in stable conditions would, therefore, learn their song more accurately and have a higher-quality song than juveniles developing in variable conditions. We further studied the relative importance of ambient conditions experienced at different ages (before vs. after hatching) and the consequences of a mismatch between them on song learning, by cross-fostering half of the hatchlings. In this case, we hypothesized that non-cross-fostered birds would learn song more accurately and develop a song of higher quality than cross-fostered siblings because the latter experienced a mismatch between the environment they were prepared for and the environment they actually developed in.

## METHODS

### Subjects and housing

We used zebra finches from a population resulting from interbreeding between the colony of the Max Planck’s Department of Behavioural Neurobiology in Seewiesen, Germany, and the colony housed at the Jagiellonian University in Kraków, Poland. We transferred 88 females and 88 males of these birds from their outdoor aviary to two indoor climatic chambers dedicated for reproduction, allowing control of day length (day/night: 13 h/11 h), humidity (60%), and temperature (see section below). Each of those chambers had 44 breeding cages (75 × 70 × 40 cm) equipped with two perches, one nest box, food (commercial mix by Megan, Poland), ad libitum water, and cuttlebone. During breeding, birds were supplemented with a mixture of boiled egg, carrots, and vitamins (Dolfos Pets, Poland) three times per week.

### Ambient conditions and breeding

**Figure 1:**
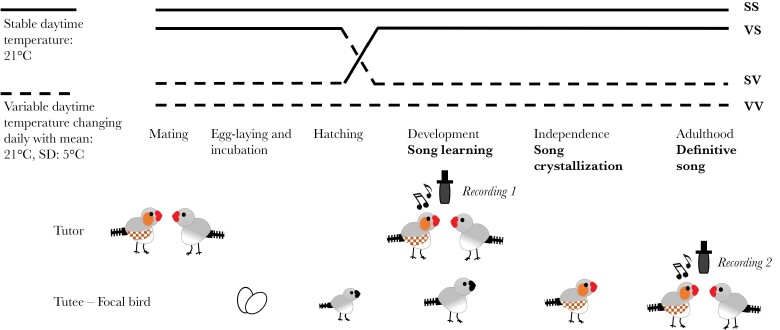
Scheme of the experimental design. Half of the juveniles in each nest were cross-fostered at hatching. The timelines of each treatment group are labeled according to their conditions during the developmental stage and song learning stage: SS (*N* = 26 males), VS (*N* = 27 males), SV (*N* = 33 males), and VV (*N* = 32 males). The song of the tutors was recorded during the song learning period of their juveniles (Recording 1) and the song of the tutees was recorded at adulthood (Recording 2).

The two climatic chambers were set to 17 °C at night and to different temperatures during daytime. The stable chamber was programmed for a constant 21 °C. In the variable chamber, the daily temperature was chosen at random following a normal distribution with a mean of 21 °C and a standard deviation of 5 °C, thereby ensuring that birds in this treatment experienced unpredictable daily temperatures ranging from 11 to 30 °C, with a mean of 21 °C (Figure 1). Dynamics of changes in the ambient temperature were similar to those encountered by zebra finches breeding in the wild, where the maximum daily temperature from day to day might differ by over 20 °C (e.g., Mariette and Buchanan 2016). This experimental setup is the same as in Lu et al. (2022).

Before breeding, male and female parents were acclimated to separate chambers for 2 months, with two birds of the same sex in each reproduction cage. Subsequently, males and females were paired and provided with nest materials while being monitored daily (Figure 1). Eggs were weighed on their laying date and labeled with a nontoxic marker. Chicks were weighed and identified at hatching by clipping a nail, and half of them in each nest were cross-fostered to the other climatic chamber, to clutches of similar size (±1 chick). At the time of cross-fostering the chicks were on average 2.09 ± SD 1.27 days old. For more details on the cross-fostering see Lu et al. (2022).

Out of 88 pairs, 5 pairs did not start breeding, 10 pairs had no offspring reaching critical song learning period, and 18 pairs were not involved in cross-fostering due to unmatching clutch size in the other chamber at hatching time. The remaining 55 pairs, which produced at least one chick that survived to independence, were involved in the analysis. In those pairs, a total of 209 chicks reached independence, including 118 males. They belonged to four treatment groups (see Figure 1): SS: Stable-Stable (54 offspring, including 26 males); VS: Variable-Stable (52 offspring, including 27 males); SV: Stable-Variable (48 offspring, including 33 males); VV: Variable-Variable (55 offspring, including 32 males). Juveniles were reared in their home cage until independence (ca. 12 weeks of age) and were then transferred to single-sex indoor aviaries with stable and variable ambient conditions matching those they experienced in their rearing chamber.

### Song recordings

We recorded the directed (courtship) song of all male tutors in a sound-proof room, with a microphone Telinga Pro 5W and the Raven Pro software (The Cornell Lab, USA, version 1.6), during the critical song learning period of their tutees (Figure 1). The temperature in the recording room was set at ca. 21–22 °C; tutors in variable chamber were recorded on days in which the temperature in the chamber was between 20 and 24 °C, and tutors in the stable chamber were recorded on the other days. One male and one unrelated female (stimulus female, other than the mate) were caught in their home cages and placed in two individual cages (70 × 30 × 40 cm) side by side in the recording room, equipped with a single perch and a water container for the female. Birds were transported between rooms in individual opaque fabric bags.

Males were placed in the recording cage, separated from the stimulus female during the preparation of the recording by a nontransparent divider (ca. 1–2 min). Recordings lasted 5 min, from the removal of the divider to the moment it was placed back between the cages. During recordings, birds were physically separated, but could see and hear each other, and the observer left the room to avoid disturbance and interference noise. Each stimulus female was used for up to eight recordings in a row, meaning a maximum of two hours in the recording room. The recording was considered successful if the male sang at least 10 renditions of his song motif, within one or more recordings. If one recording was not enough, the male was recorded again later during the day or the week with another stimulus female. We proceeded until all the tutors were successfully recorded.

We used the same protocol to record the song of all male offspring at adulthood (i.e., after 150 days of age). However, due to a very low propensity to sing during the first recording sessions, we established a habituation period during which male tutees (i.e., adult offspring) were randomly caught in their aviary and placed in same-sex pairs in four cages identical to the recording cage but in different sound-proof room. Habituation lasted from 1.5 to 4 h prior to recording, food and water were provided ad libitum. Birds were given at least 10 trials before we stopped attempts to record at least 10 renditions of their song. If unsuccessful, we attributed a 0 in their propensity to sing (see Statistical analysis).

### Song analysis

Song recordings were analyzed in Raven Pro 1.6. We measured the song rate (duration of all the song bouts produced divided by the total duration of the recordings in which singing occurred) and the number of song motifs produced per 5 min (duration of the recordings), and we noted the number of recording trials before the first successful recording.

We analyzed the recordings at the syllable level, which is considered to be the most biologically relevant element of zebra finch song (Holveck and Riebel 2007). We defined syllables as song elements separated by at least 5 ms of silence. Using this criterion, exemplars of each syllable type were first automatically detected with Raven for each male and identifications were then manually confirmed by a human observer. The first 10 clear renditions of each syllable were subsequently selected for analysis, labeled, and saved separately. As song quality features, we measured the syllable repertoire size and the syllable consistency. The identification of the number of unique syllable types allowed us to determine the syllable repertoire size of each bird. As not all males used introductory notes, we reported the absence or presence of these notes as a variable. Zebra finches learn their song by copying the song of their tutors. Accordingly, we assessed the quality of learning by measuring song learning accuracy as the resemblance between tutors’ and tutees’ shared syllables. Quantity of learning was assessed by calculating the proportion of the tutor’s syllable repertoire that has been copied by his tutees.

We created spectral cross-correlation matrices with the 10 renditions of each syllable of each male to compute their similarity scores and measured the syllable consistency. These scores range between 0 (lack of similarity) and 1 (perfect similarity, identical). We generated the matrices in Raven and imported them to R v4.0.3 (R Core Team 2020) for analysis, using the packages Rraven and warbleR (Araya-Salas and Smith-Vidaurre 2017; Araya-Salas 2020). We considered song learning accuracy as the similarity score between syllables of the tutee and the corresponding syllables of his tutor. To compute this score, we created a global cross-correlation matrix including every syllable of every bird (tutors and tutees). For each pair of tutor and tutee, the program automatically associated the syllables with the highest similarity score and reported this value as our learning accuracy metric. To avoid mistakes induced by bad learning or improvisation, we verified those associations visually. Whenever we observed disagreements between automatic and visual methods, we removed such syllables from the analysis.

### Statistical analysis

All the analyses were performed in R v4.0.3 (R Core Team 2020). To test the potential effect of temperature conditions before and after hatching and their interaction on the propensity to sing of the tutees, we used a Mixed effects Cox regression using the packages survival and coxme (Therneau and Grambsch 2000; Therneau 2012, 2023). The model analyzed the probability to sing of the birds based on the recording success (event) and the number of trials (time). We then calculated both tutors and tutees’ motivation to sing, using principal component analysis (PCA). The PCA included the number of trials before singing, the number of song bouts produced, and the song rate (=duration of singing behavior over recording duration), which were log-transformed to normalize their distribution. In the case of tutees, the first component extracted from the data set accounted for 72.9% of the total variance. The three variables had the following loadings: number of trials before singing: −0.49, number of song bouts: 0.60, song rate: 0.62. The component was described as “tutees motivation.” In the case of tutors, the first component extracted from the data set explained 71.4% of the total variance. The three variables had the following loadings: number of trials before singing: −0.39, number of song bouts: 0.64, song rate: 0.66. The component was described as “tutors motivation.”

We analyzed the syllable repertoire size, the mean and maximum syllable consistency, the song learning accuracy, and the proportion of copied syllables using linear mixed models (LMM, Gaussian error distribution) with package lme4 (Bates et al. 2014). To analyze the presence or absence of introductory notes we used a GLMM with binomial error distribution and logit link. In each case, the models included the temperature treatment before and after hatching, and the interaction of those two factors, tutors and tutee motivation to sing (as detailed above), the number of male tutees in the nest and the performance of the tutor for the same measure (repertoire size, syllable mean, and maximum consistency). We verified that tutor repertoire size did not differ between the four experimental groups and we used it as a covariate in the models testing tutee learning (learning accuracy and proportion of copied syllables from the tutor).

The tutor ID and the female ID used as stimuli during recording were included in the models as random effects. Random effect of tutee ID was included in the analyses of learning accuracy, which was carried out at the level of syllable. We verified the assumptions of all the models by visualizing the distribution of the residuals and checking their normality and homoscedasticity. The figures presented in this article were generated using the ggplot2 and survminer packages (Wickham 2016; Kassambara et al. 2021).

### Ethical note

This study was authorized by the 2nd Local Institutional Animal Care and Use Committee (IACUC) in Cracow, Poland; permit no. 155/2019. Birds were housed and bred following the European Union Law for experimentation with animals. Birds had access to food and water ad libitum and were monitored daily.

## RESULTS

### Participation and sample size

**Figure 2:**
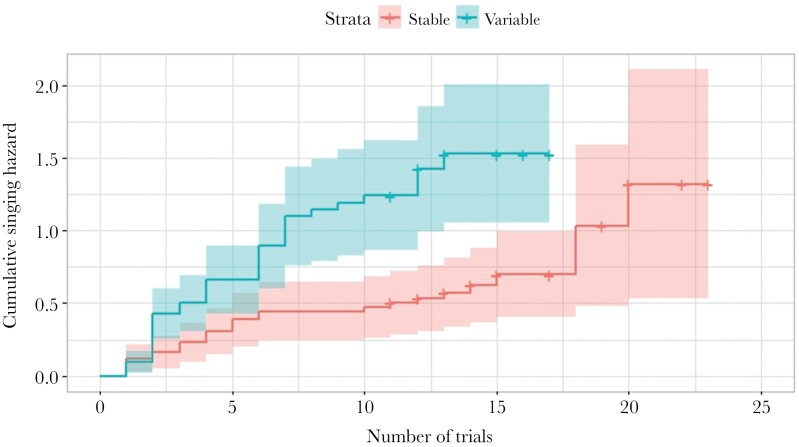
Propensity to sing of the tutees according to the temperature conditions after hatching. The propensity to sing is expressed as the “cumulative hazard” probability of the birds singing during recordings. The colors correspond to the conditions after hatching (red: stable; blue: variable). The colored areas represent the 95% confidence interval. Crosses correspond to the maximum number of trials of the nonsinging birds.

Fifty-five tutors and their 118 male tutees were involved in this study, of which all tutors and 76 tutees were successfully recorded. The analysis of the propensity to sing showed a significant effect of the temperature conditions after hatching on the probability of singing (χ^2^(1) = 7.993, *P* = 0.005) but no effect of the condition before hatching (χ^2^(1) = 1.931, *P* = 0.165) nor of the interaction between those two periods (χ^2^(1) = 0.642, *P* = 0.423). The effect of tutor ID was not significant (comparison of models with and without random effects, χ^2^(1) = 0.018, *P* = 0.893). Tutees developing in variable conditions were more likely to sing (Figure 2). Only the males which produced at least 10 song motifs are included in the following results. The sample sizes are as follows: SS group: 12 tutees from 10 tutors; SV: 23 tutees from 16 tutors; VS: 16 tutees from 16 tutors; VV: 25 tutees from 17 tutors.

### Syllable repertoire size

The syllable repertoire size of the tutees was not affected by the temperature treatment at any stage (Tables 1 and 2). Instead, the syllable repertoire size of the tutees depended on the one of their tutor (Table 2). We also observed that the syllable repertoire size was negatively affected by the number of males in the brood and by the motivation of their tutor (Table 2). The analysis of the presence of introductory notes revealed no relationship with any of the factors or covariates.

**Table 1:** Song learning and quality measures in the four experimental groups

Song measures	SS	SV	VS	VV
Syllable repertoire size	4.76 ± 0.34	4.17 ± 0.25	4.29 ± 0.29	4.51 ± 0.24
Mean syllable consistency	0.637 ± 0.034	0.645 ± 0.027	0.661 ± 0.030	0.674 ± 0.027
Maximum syllable consistency	0.796 ± 0.049	0.698 ± 0.048	0.739 ± 0.047	0.786 ± 0.046
Syllable learning accuracy	0.367 ± 0.019	0.368 ± 0.015	0.394 ± 0.019	0.406 ± 0.014
Proportion of copied syllables (%)	74.63 ± 5.62	69.38 ± 4.07	67.69 ± 4.83	73.75 ± 3.81

The marginal means and standard errors from the models are presented.

**Table 2:** Results of the LMM in which the syllable repertoire size of the tutees was the response variable

Fixed effects	Estimate (SE)	df	*F*	*P*
Before hatching	−0.371 (0.394)	65.549	0.003	0.957
After hatching	−0.544 (0.395)	67.542	0.435	0.512
Before × After hatching	0.714 (0.495)	64.850	2.079	0.154
Tutees motivation	−0.005 (0.084)	67.901	0.003	0.955
Tutors motivation	−0.208 (0.086)	64.951	5.839	0.018
Syllable repertoire size tutor	0.304 (0.073)	67.718	17.124	9.90e−05
Number of males in the brood	−0.507 (0.155)	67.773	10.690	0.002

Random effects		Variance	SD	*P*
Tutor ID		0.000	0.000	1.000
Female ID		0.053	0.230	0.483

The estimates with standard error are reported with the results of analysis of variance.

### Syllable consistency

As syllable consistency measures, we reported the mean of the similarity scores for each male, as well as the highest syllable mean similarity score for each male (maximum consistency, Table 1). Neither the mean nor the maximum syllable consistency was affected by the ambient conditions. The motivation component of the tutees affected the maximum consistency, which was higher when the male was singing more (Table 3).

**Table 3:** Results of the LMM in which the mean or maximum syllable consistency of the tutees were the response variables

	Mean syllable consistency				Maximum syllable consistency			
Fixed effects	Estimate (SE)	df	*F*	*P*	Estimate (SE)	df	*F*	*P*
Before hatching	0.027 (0.033)	32.128	1.796	0.190	−0.007 (0.032)	43.279	0.536	0.468
After hatching	0.011 (0.035)	32.393	0.219	0.643	−0.022 (0.038)	35.857	0.001	0.976
Before × After hatching	0.003 (0.042)	34.824	0.004	0.951	0.043 (0.039)	47.911	1.193	0.280
Tutees motivation	0.011 (0.008)	62.362	2.188	0.144	0.014 (0.008)	56.426	3.242	0.077
Tutors motivation	−0.005 (0.008)	20.110	0.376	0.547	−0.015 (0.010)	32.875	2.425	0.129
Mean/max consistency tutor	0.147 (0.104)	28.192	1.973	0.171	0.056 (0.107)	40.003	0.274	0.604
Number of males in the brood	0.003 (0.014)	34.545	0.039	0.845	−0.003 (0.016)	37.990	0.031	0.861

Random effects		Variance	SD	*P*		Variance	SD	P
Tutor ID		0.001	0.025	0.744		0.004	0.066	0.008
Female ID		0.002	0.048	0.198		0.012	0.111	0.002

The estimates with standard error are reported with the results of analysis of variance.

### Song learning

**Figure 3:**
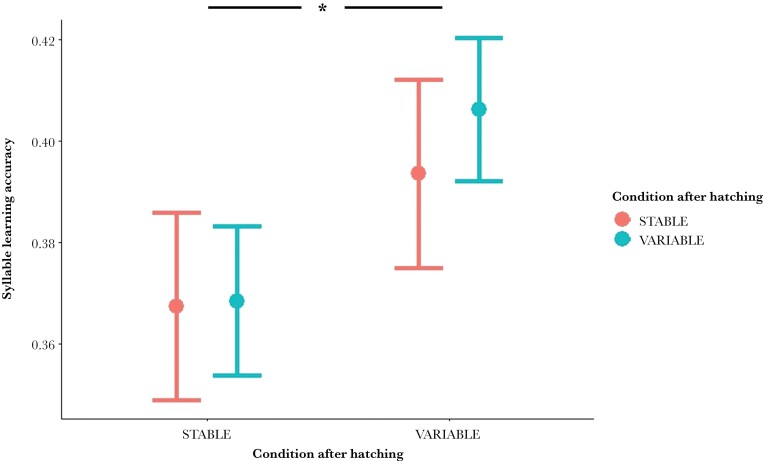
Syllable learning accuracy of the tutees according to the temperature conditions before and after hatching. The learning accuracy is expressed as the similarity scores (between 0 and 1) between the syllables of the tutees and the corresponding syllables of their tutor. The colors correspond to the conditions after hatching (red: stable; blue: variable). Marginal effects of the conditions Before × After hatching interaction are reported, and the bars represent the standard error. Asterisk indicates significant difference between the two conditions experienced before hatching.

The syllable learning accuracy was affected by the condition before, but not after hatching (Tables 1 and 4). Males originating from variable temperatures condition before hatching learned the syllables of their tutor more accurately than those originating from stable temperatures condition, regardless of which conditions they were reared in after birth (Figure 3). The motivation of the tutor also had a positive impact on the learning accuracy (Table 4). The proportion of the tutor’s syllables that was copied was not affected by our treatment (Table 4). This learning aspect was negatively affected by the repertoire size of the tutor, more syllables to copy from the tutor decreased the proportion that has actually been copied.

**Table 4:** Results of the LMM in which the learning measures (syllable learning accuracy and proportion of copied syllables from the tutor) of the tutees were the response variable

	Syllable learning accuracy				Proportion of copied syllables			
Fixed effects	Estimate (SE)	df	*F*	*P*	Estimate (SE)	df	*F*	*P*
Before hatching	0.024 (0.024)	250.52	4.005	0.046	−6.859 (7.010)	66.368	0.082	0.776
After hatching	0.001 (0.023)	239.35	0.205	0.651	−5.073 (7.305)	38.538	0.010	0.921
Before × After hatching	0.013 (0.030)	252.91	0.198	0.657	11.204 (8.746)	66.442	1.641	0.205
Tutees motivation	0.007 (0.005)	189.41	1.694	0.195	0.419 (1.480)	68.000	0.080	0.779
Tutors motivation	0.017 (0.005)	254.62	10.682	0.001	−1.383 (1.674)	28.191	0.682	0.416
Syllable repertoire size tutor	0.007 (0.004)	221.02	2.240	0.136	−4.618 (1.406)	28.314	10.790	0.003
Number of males in the brood	0.007 (0.009)	232.71	0.640	0.424	−4.718 (2.901)	38.398	2.645	0.112

Random effects		Variance	SD	*P*		Variance	SD	P
Tutor ID		0.000	0.000	1.000		44.65	6.682	0.413
Female ID		<0.001	0.016	0.313		0.000	0.000	1.000
Tutee ID		0.000	0.000	1.000				

The estimates with standard error are reported with the results of analysis of variance.

## DISCUSSION

We investigated the potential impact of variable ambient temperature at both short and long timescales on a zebra finch’s ability to learn and display song, a critically important trait for fitness in this species. In our study, we found that variable temperatures experienced before hatching promotes more accurate learning of syllables (Figure 3), and that variable temperatures experienced after hatching induce male singing (Figure 2). Neither the proportion of syllables copied from a tutor nor the syllable repertoire size were affected by temperature variation at any stage. Similarly, a mismatch between conditions before and after hatching had no impact on any of the song parameters measured. Taken together, these results indicate that, in contrast to our predictions, temperature variability, experienced as its daily changes or as an alteration of conditions after hatching, did not lead to cognitive impairment, but had a positive impact on song learning and performance in the zebra finch.

### Participation

Because singing is the first step of courtship in zebra finches (Zann 1996), birds that either rarely sing or do not sing at all are unlikely to mate. During our recordings, 35% of the tutees did not produce a song. Those males were distributed in all treatment groups, although tutees reared in stable conditions were less likely to sing than tutees reared in variable conditions (Figure 2). In many studies, birds are recorded until they eventually sing, or nonsinging individuals are removed from the analysis; so information about the number of nonsingers and their distribution in different treatment groups is rare in the literature (Schmidt et al. 2013; Honarmand et al. 2015). When other studies aimed to consider participation in recordings, they measured the delay between the beginning of the recording and the first song produced. This latency was either longer in birds experiencing nutritional stress (Buchanan et al. 2003; Ritschard and Brumm 2012) or not affected by treatment (Kriengwatana et al. 2014). However, singing latency reveals more the motivation of an individual, than its actual participation, which is a binary variable (i.e., did or did not participate). Participation using the latter definition has been accounted for in an associative learning test (Lu et al. 2022). Using the same study design and the same birds as presented here, Lu et al. (2022) found a higher participation rate in birds reared in variable conditions compared with the other groups. Variable conditions made the birds more active in foraging tasks, and seems, to a lower extent, to make the males more active in courtship.

### Syllable repertoire size

Syllable repertoire size is frequently viewed as a measure of male quality because more complex songs tend to be favored by females (reviewed in Riebel 2009). It is also widely assumed that repertoire size reflects early developmental effects (Nowicki et al. 2002; Spencer et al. 2003, 2005; Riebel 2009). We expected that ambient temperature may affect this song trait, as Zann and Cash (2008) found seasonal effects on the syllable repertoire size in wild zebra finches. Specifically, juveniles reared in autumn had a larger syllable repertoire size than those reared in winter. Surprisingly, in our study, neither the temperature variability nor the mismatch between conditions before and after hatching affected syllable repertoire size. In contrast, repertoire size was negatively correlated with the number of males in the brood (Table 2), as has been previously shown in other studies (Tchernichovski and Nottebohm 1998; Gil et al. 2006; Soma et al. 2006).

The repertoire size in the zebra finch has been shown to reflect early developmental stress. In some studies, song quality was affected by corticosterone treatment, food deprivation (Spencer et al. 2003, 2005; Buyannemekh et al. 2020), and brood size (Tchernichovski and Nottebohm 1998; Gil et al. 2006; Soma et al. 2006). However, other studies (Zann and Cash 2008; Brumm et al. 2009; Ritschard and Brumm 2012; Kriengwatana et al. 2014) found no effect of food restriction during development on the syllable repertoire size in zebra finches. Our results show that temperature variability may not be a stressor severe enough to impair song complexity.

### Syllable consistency

Zebra finches are capable of perceiving slight variations in temporal fine structure that can provide information on a male’s current and past environment and physiological condition (Dooling and Prior 2017) and can therefore be useful in female choice. In our study, neither the mean nor the maximum syllable consistency were significantly affected by temperature treatment. It is possible that syllable consistency may be related to conditions at the time of singing (Schmidt et al. 2014). We recorded all males at the same temperature, and we recorded birds from the variable conditions only when the temperature in their chamber matched the temperature in the recording room. Thus, all the birds were recorded under the same conditions, which could explain the observed similarities between treatment groups.

### Song learning

We observed higher syllable learning accuracy in birds that originated from variable condition before hatching, regardless of the condition in which they were subsequently reared (Table 4 and Figure 3). However, we found no effect of our treatment on the proportion of syllables copied from the tutor (Table 4). These results show that temperature variability, especially before hatching, influences quality but not quantity of song learning. Cross-fostering of the chicks at hatching allowed us to determine which developmental period is the most likely to influence learning accuracy and highlight potential parental effects. To our knowledge, this is the first evidence of a significant impact of prenatal conditions on song learning.

It has previously been shown that egg laying order affects learning accuracy (Tchernichovski and Nottebohm 1998) and song syntax, but not syllable repertoire size (Soma et al. 2009). Similarly, hatching asynchrony is known to influence spatial and motor learning but not song learning accuracy in zebra finches (Campbell et al. 2017). Although we found no difference in egg mass between treatment groups, we acknowledge that other parental effects such as epigenetic effects (Guerrero-Bosagna et al. 2018), sperm quality, egg composition, breeding behavior, and parental vocalizations (Katsis et al. 2018), could have shaped the cognitive abilities of the offspring as well. However, it is unlikely that the temperature in the nest had been a factor of this variation, since parent zebra finches have been shown to maintain a similar nest temperature in cold and hot ambient conditions (Ton et al. 2021). Our study does not allow us to conclude which mechanism(s) enhance later learning, and we are unable to tell if temperature conditions were more determining before breeding, during egg laying, during incubation, or at some or all of these stages. Further investigation of parental effects could bring valuable evidence on the development of learning abilities and song structure.

It is perhaps most surprising that variable ambient conditions before hatching enhanced learning accuracy, because prenatal maternal stress has been shown to have a detrimental impact on the learning abilities of the offspring in mammals (see Weinstock 2008 for a review). However, another study on fish demonstrated that offspring can compensate for a learning deficit by using more social cues (Feng et al. 2015). In zebra finches, social interactions are necessary for an efficient song learning (Carouso-Peck et al. 2020), and juveniles from variable prehatching condition in our study may have relied more on these interactions than those from stable prehatching condition, although this hypothesis would need further investigation. Another explanation could lay in the fact that variable temperature may not be perceived as a stressor, but on the contrary could be an enrichment, as suggested by Lu et al. (2022). Indeed, the variable condition resembles more the natural habitat of zebra finches than the stable condition (Zann 1996; Mariette and Buchanan 2016) and could explain the higher learning performance we found in our study. Also at a comparative scale, the unpredictability of temperature and precipitation were correlated with a more complex and consistent song and higher song rate in Mimidae (Botero et al. 2009).

### Absence of mismatch effect

Contrary to our predictions, a mismatch between conditions before and after hatching did not impact any of our measures of song learning and production. Such effect would have been visible as a significant interaction term of temperature conditions before and after hatching. Other studies showed the negative impact of a mismatch in environmental conditions during development, including temperature (Costantini et al. 2014), on birds’ phenotype and physiology (Giordano et al. 2014; Krause and Naguib 2014). However, a given value of a trait such as size or mass, can be adaptive in one situation and unfavorable in the opposite situation. On the contrary, song performance is a quality trait only when it is high, and song learning abilities may result from a developmental trade-off rather than adaptive programming (MacDougall-Shackleton 2015).

## CONCLUSION

In this study, we investigated the impact of ambient temperature variability on song learning and quality, at different developmental stages (i.e., before and after hatching). Contrary to our expectations, variable ambient temperatures did not impact syllable repertoire size or the proportion of syllables copied from a tutor. This result could indicate that temperature variability might not impose a challenge sufficiently severe to disturb song development. Instead, this variable temperature mimicking natural conditions could act as an environmental enrichment. This would explain the higher propensity to sing in birds raised under variable conditions and the higher syllable learning accuracy observed in birds that originated from variable temperature conditions prior to hatching. We have shown experimentally and for the first time, that prehatching variability and predictability of the environment can have significant effects on song learning accuracy. There is a possibility that parental effects contributed to this effect, although their nature and the extent to which they can influence song learning of the offspring remain to be determined.

## Notes

We want to thank Marzena Majgier and Sylwester Kunysz for their time and help with the birds, and Anni Hämäläinen, Marta Grosiak, Alaa Hseiky, and Kyle Coughlan who helped improving the manuscript and Mariusz Cichoń for support. We also thank the Department of Behavioural Neurobiology in Seewiesen for the birds they provided. We finally thank the three anonymous referees and the editor Louise Barrett, who provided insightful comments on a previous draft of this article and helped improving it a lot.

## References

[CIT0001] Acevedo-Whitehouse K , Duffus ALJ. 2009. Effects of environmental change on wildlife health. Philos Trans R Soc Lond B Biol Sci. 364:3429–3438. 19833653 10.1098/rstb.2009.0128 PMC2781848

[CIT0002] Araya-Salas M. 2020. Rraven: connecting R and Raven bioacoustic software (R package version 1.0.9).

[CIT0003] Araya-Salas M , Smith-Vidaurre G. 2017. WarbleR: an r package to streamline analysis of animal acoustic signals. Methods Ecol Evol. 8:184–191.

[CIT0004] Bates D , Mächler M, Bolker B, Walker S. 2014. Fitting linear mixed-effects models using lme4. J Stat Softw. 67(1):1–48. doi:10.18637/jss.v067.i01.

[CIT0005] Bertin A , Richard-Yris M-A, Möstl E, Lickliter R. 2009. Increased yolk testosterone facilitates prenatal perceptual learning in Northern bobwhite quail (*Colinus virginianus*). Horm Behav. 56:416–422. 19646986 10.1016/j.yhbeh.2009.07.008

[CIT0006] Botero CA , Boogert NJ, Vehrencamp SL, Lovette IJ. 2009. Climatic patterns predict the elaboration of song displays in mockingbirds. Curr Biol. 19:1151–1155. 19464180 10.1016/j.cub.2009.04.061 PMC3541702

[CIT0007] Briga M , Verhulst S. 2015. Large diurnal temperature range increases bird sensitivity to climate change. Sci Rep. 5:16600. doi:10.1038/srep16600 26563993 PMC4643245

[CIT0008] Brumm H , Zollinger SA, Slater PJB. 2009. Developmental stress affects song learning but not song complexity and vocal amplitude in zebra finches. Behav Ecol Sociobiol. 63:1387–1395. 19554102 10.1007/s00265-009-0749-y PMC2699386

[CIT0009] Buchanan KL , Spencer KA, Goldsmith AR, Catchpole CK. 2003. Song as an honest signal of past developmental stress in the European starling (*Sturnus vulgaris*). Proc R Soc Lond B Biol Sci. 270:1149–1156. 10.1098/rspb.2003.2330 PMC1691349 12816653

[CIT0010] Buyannemekh K , Zito JB, Tomaszycki ML. 2020. Early life nutritional stress affects song learning but not underlying neural circuitry in zebra finches. Behav Neurosci. 134(3):222–232. doi:10.1037/bne0000362 32223278

[CIT0011] Campbell SA , Beck ML, Sewall KB. 2017. Hatching asynchrony impacts cognition in male zebra finches. J Exp Zool A Ecol Integr Physiol. 327:89–97. 28544577 10.1002/jez.2074

[CIT0012] Carouso-Peck S , Menyhart O, DeVoogd TJ, Goldstein MH. 2020. Contingent parental responses are naturally associated with zebra finch song learning. Anim Behav. 165:123–132.

[CIT0013] Cohen J , Agel L, Barlow M, Garfinkel CI, White I. 2021. Linking Arctic variability and change with extreme winter weather in the United States. Science. 373(6559):1116–1121. doi:10.1126/science.abi9167 34516838

[CIT0014] Colson V , Cousture M, Damasceno D, Valotaire C, Nguyen T, Cam AL, Bobe J. 2019. Maternal temperature exposure impairs emotional and cognitive responses and triggers dysregulation of neurodevelopment genes in fish. PeerJ. 7:e6338. 30723624 10.7717/peerj.6338 PMC6360074

[CIT0015] Coomes CM , Derryberry EP. 2021. High temperatures reduce song production and alter signal salience in songbirds. Anim Behav. 180:13–22.

[CIT0016] Costantini D , Monaghan P, Metcalfe NB. 2014. Prior hormetic priming is costly under environmental mismatch. Biol Lett. 10:20131010. 24522630 10.1098/rsbl.2013.1010 PMC3949371

[CIT0017] Coumou D , Rahmstorf S. 2012. A decade of weather extremes. Nat Clim Change. 2:491–496.

[CIT0018] Couriot O , Hewison AJM, Saïd S, Cagnacci F, Chamaillé-Jammes S, Linnell JDC, Mysterud A, Peters W, Urbano F, Heurich M, et al. 2018. Truly sedentary? The multi-range tactic as a response to resource heterogeneity and unpredictability in a large herbivore. Oecologia. 187:47–60. 29610976 10.1007/s00442-018-4131-5

[CIT0019] Dooling RJ , Prior NH. 2017. Do we hear what birds hear in birdsong? Anim Behav. 124:283–289. 29628517 10.1016/j.anbehav.2016.10.012 PMC5884127

[CIT0020] Dueñas A , Jiménez-Uzcátegui G, Bosker T. 2021. The effects of climate change on wildlife biodiversity of the Galapagos Islands. Clim Change Ecol. 2:100026.

[CIT0021] Eaton L , Edmonds EJ, Henry TB, Snellgrove DL, Sloman KA. 2015. Mild maternal stress disrupts associative learning and increases aggression in offspring. Horm Behav. 71:10–15. 25840012 10.1016/j.yhbeh.2015.03.005

[CIT0022] Feng S , McGhee KE, Bell AM. 2015. Effect of maternal predator exposure on the ability of stickleback offspring to generalize a learned colour–reward association. Anim Behav. 107:61–69. 29046591 10.1016/j.anbehav.2015.05.024 PMC5642945

[CIT0023] Forstmeier W , Coltman DW, Birkhead TR. 2004. Maternal effects influence the sexual behavior of sons and daughters in the zebra finch. Evolution. 58:2574–2583. 15612299 10.1111/j.0014-3820.2004.tb00885.x

[CIT0024] Gil D , Naguib M, Riebel K, Rutstein A, Gahr M. 2006. Early condition, song learning, and the volume of song brain nuclei in the zebra finch (*Taeniopygia guttata*). J Neurobiol. 66:1602–1612. 17058194 10.1002/neu.20312

[CIT0025] Giordano M , Groothuis TGG, Tschirren B. 2014. Interactions between prenatal maternal effects and posthatching conditions in a wild bird population. Behav Ecol. 25:1459–1466.

[CIT0026] Glądalski M , Bańbura M, Kaliński A, Markowski M, Skwarska J, Wawrzyniak J, Zieliński P, Bańbura J. 2018. Hatching delays in great tits and blue tits in response to an extreme cold spell: a long-term study. Int J Biometeorol. 62:1437–1445. 29667035 10.1007/s00484-018-1541-3 PMC6063324

[CIT0027] Griffith SC , Buchanan KL. 2010. Maternal effects in the Zebra Finch: a model mother reviewed. Emu. 110:251–267.

[CIT0028] Groothuis TGG , Müller W, von Engelhardt N, Carere C, Eising C. 2005. Maternal hormones as a tool to adjust offspring phenotype in avian species. Neurosci Biobehav Rev. 29:329–352. 15811503 10.1016/j.neubiorev.2004.12.002

[CIT0029] Groothuis TGG , Taborsky B. 2015. Introducing biological realism into the study of developmental plasticity in behaviour. Front Zool. 12:S6. 26816523 10.1186/1742-9994-12-S1-S6 PMC4722348

[CIT0030] Guerrero-Bosagna C , Morisson M, Liaubet L, Rodenburg TB, de Haas EN, Košťál Ľ, Pitel F. 2018. Transgenerational epigenetic inheritance in birds. Environ Epigenet. 4:dvy008. 29732172 10.1093/eep/dvy008 PMC5920295

[CIT0031] Holveck MJ , Riebel K. 2007. Preferred songs predict preferred males: consistency and repeatability of zebra finch females across three test contexts. Anim Behav. 74:297–309.

[CIT0032] Honarmand M , Riebel K, Naguib M. 2015. Nutrition and peer group composition in early adolescence: impacts on male song and female preference in zebra finches. Anim Behav. 107:147–158.

[CIT0033] Kassambara A , Kosinski M, Biecek P, Fabian S. 2021. survminer: drawing survival curves using “ggplot2” (0.4.9). https://rpkgs.datanovia.com/survminer/index.html [Accessed on 2023 Jan 20].

[CIT0034] Katsis AC , Davies MH, Buchanan KL, Kleindorfer S, Hauber ME, Mariette MM. 2018. Prenatal exposure to incubation calls affects song learning in the zebra finch. Sci Rep. 8:15232. 10.1038/s41598-018-33301-5 30323211 PMC6189107

[CIT0035] Krause ET , Naguib M. 2014. Effects of parental and own early developmental conditions on the phenotype in zebra finches (*Taeniopygia guttata*). Evol Ecol. 28:263–275.

[CIT0036] Kriengwatana B , Wada H, Schmidt KL, Taves MD, Soma KK, MacDougall-Shackleton SA. 2014. Effects of nutritional stress during different developmental periods on song and the hypothalamic–pituitary–adrenal axis in zebra finches. Horm Behav. 65:285–293. 24417905 10.1016/j.yhbeh.2013.12.013

[CIT0037] Lefeuvre M , Lu C, Botero CA, Rutkowska J. 2023. Variable ambient temperature promotes song learning and production in zebra finches. Behav Ecol. doi:10.5061/dryad.bvq83bkdz PMC10183203 37192924

[CIT0038] Lindström J. 1999. Early development and fitness in birds and mammals. Trends Ecol Evol. 14:343–348. 10441307 10.1016/s0169-5347(99)01639-0

[CIT0039] London SE. 2019. Developmental song learning as a model to understand neural mechanisms that limit and promote the ability to learn. Behav Processes. 163:13–23. 29162376 10.1016/j.beproc.2017.11.008

[CIT0040] Lu C , Lefeuvre M, Rutkowska J. 2022. Variability in ambient temperature promotes juvenile participation and shorter latency in a learning test in zebra finches. Anim Behav. 186:57–66.

[CIT0041] MacDougall-Shackleton SA. 2015. Developmental stress and birdsong: integrating signal function and development. Curr Opin Behav Sci. 6:104–110.

[CIT0042] Mann NI , Slater PJB. 1995. Song tutor choice by zebra finches in aviaries. Anim Behav. 49:811–820. 10.1006/anbe.1998.0924 10053084

[CIT0043] Mariette MM , Buchanan KL. 2016. Prenatal acoustic communication programs offspring for high posthatching temperatures in a songbird. Science. 353:812–814. 27540172 10.1126/science.aaf7049

[CIT0044] McCarty R. 2017. Cross-fostering: elucidating the effects of gene × environment interactions on phenotypic development. Neurosci Biobehav Rev. 73:219–254. 28034661 10.1016/j.neubiorev.2016.12.025

[CIT0045] Milligan SR , Holt WV, Lloyd R. 2009. Impacts of climate change and environmental factors on reproduction and development in wildlife. Philos Trans R Soc Lond B Biol Sci. 364:3313–3319. 19833643 10.1098/rstb.2009.0175 PMC2781851

[CIT0046] Monaghan P. 2008. Early growth conditions, phenotypic development and environmental change. Philos Trans R Soc Lond B Biol Sci. 363:1635–1645. 18048301 10.1098/rstb.2007.0011 PMC2606729

[CIT0047] Mousseau TA , Fox CW. 1998. Maternal effects as adaptations. Oxford: Oxford University Press.

[CIT0048] Müller W , Eens M. 2009. Elevated yolk androgen levels and the expression of multiple sexually selected male characters. Horm Behav. 55:175–181. 18976657 10.1016/j.yhbeh.2008.09.012

[CIT0049] Müller W , Vergauwen J, Eens M. 2008. Yolk testosterone, postnatal growth and song in male canaries. Horm Behav. 54:125–133. 18353330 10.1016/j.yhbeh.2008.02.005

[CIT0050] Munn AJ , Kern P, McAllan BM. 2010. Coping with chaos: unpredictable food supplies intensify torpor use in an arid-zone marsupial, the fat-tailed dunnart (*Sminthopsis crassicaudata*). Naturwissenschaften. 97:601–605. 20442980 10.1007/s00114-010-0670-2

[CIT0051] Naguib M , Nemitz A, Gil D. 2006. Maternal developmental stress reduces reproductive success of female offspring in zebra finches. Proc R Soc B Biol Sci. 273:1901–1905. 10.1098/rspb.2006.3526 PMC1634771 16822750

[CIT0052] Nord A , Giroud S. 2020. Lifelong effects of thermal challenges during development in birds and mammals. Front Physiol. 11:419. doi:10.3389/fphys.2020.00419 PMC7261927 32523540

[CIT0053] Nowicki S , Searcy W, Peters S. 2002. Brain development, song learning and mate choice in birds: a review and experimental test of the “nutritional stress hypothesis”. J Comp Physiol A. 188:1003–1014. 10.1007/s00359-002-0361-3 12471497

[CIT0054] Popic TJ , Wardle GM. 2006. Extremes: understanding flower-visitor interactions in a changing climate. In: Lunney D, Pat H, editors. Wildlife and climate change: towards robust conservation strategies for Australian fauna. New South Wales, Australia: Royal Zoological Society of New South Wales. p. 99–106.

[CIT0055] R Core Team. 2020. R: a language and environment for statistical computing. R Foundation for Statistical Computing. https://www.R-project.org/ [Accessed on 2022 June 11].

[CIT0056] Raffel TR , Romansic JM, Halstead NT, McMahon TA, Venesky MD, Rohr JR. 2013. Disease and thermal acclimation in a more variable and unpredictable climate. Nat Clim Change. 3:146–151.

[CIT0057] Remacle C , Bieswal F, Bol V, Reusens B. 2011. Developmental programming of adult obesity and cardiovascular disease in rodents by maternal nutrition imbalance. Am J Clin Nutr. 94(Suppl 6):1846S–1852S. 21543546 10.3945/ajcn.110.001651

[CIT0058] Riebel K. 2009. Chapter 6 Song and female mate choice in zebra finches: a review. In: Naguib M, Zuberbuumlhler K, Clayton NS, Janik VM, editors. Advances in the study of behavior, Vol. 40. Elsevier, Amsterdam: Academic Press. p. 197–238.

[CIT0059] Ritschard M , Brumm H. 2012. Zebra finch song reflects current food availability. Evol Ecol. 26:801–812.

[CIT0060] Roper A , Zann R. 2006. The onset of song learning and song tutor selection in fledgling zebra finches. Ethology. 112:458–470.

[CIT0061] Roth TC , Rattenborg NC, Pravosudov VV. 2010. The ecological relevance of sleep: the trade-off between sleep, memory and energy conservation. Philos Trans R Soc Lond B Biol Sci. 365:945–959. 20156818 10.1098/rstb.2009.0209 PMC2830243

[CIT0062] Rutkowska J , Lagisz M, Bonduriansky R, Nakagawa S. 2020. Mapping the past, present and future research landscape of paternal effects. BMC Biol. 18:183. 10.1186/s12915-020-00892-3 33246472 PMC7694421

[CIT0063] Ruuskanen S , Groothuis TGG, Schaper SV, Darras VM, de Vries B, Visser ME. 2016. Temperature-induced variation in yolk androgen and thyroid hormone levels in avian eggs. Gen Comp Endocrinol. 235:29–37. 27255366 10.1016/j.ygcen.2016.05.026

[CIT0064] Saalfeld ST , McEwen DC, Kesler DC, Butler MG, Cunningham JA, Doll AC, English WB, Gerik DE, Grond K, Herzog P, et al. 2019. Phenological mismatch in Arctic-breeding shorebirds: impact of snowmelt and unpredictable weather conditions on food availability and chick growth. Ecol Evol. 9:6693–6707. 31236253 10.1002/ece3.5248 PMC6580279

[CIT0065] Sattar Q , Maqbool ME, Ehsan R, Akhtar S, Sattar Q, Maqbool ME, Ehsan R, Akhtar S. 2021. Review on climate change and its effect on wildlife and ecosystem. Open J Environ Biol. 6:8–14.

[CIT0066] Schmidt KL , MacDougall-Shackleton EA, Kubli SP, MacDougall-Shackleton SA. 2014. Developmental stress, condition, and birdsong: a case study in song sparrows. Integr Comp Biol. 54:568–577. 24951504 10.1093/icb/icu090

[CIT0067] Schmidt KL , Moore SD, MacDougall-Shackleton EA, MacDougall-Shackleton SA. 2013. Early-life stress affects song complexity, song learning and volume of the brain nucleus RA in adult male song sparrows. Anim Behav. 86:25–35.

[CIT0068] Schoech SJ , Rensel MA, Heiss RS. 2011. Short- and long-term effects of developmental corticosterone exposure on avian physiology, behavioral phenotype, cognition, and fitness: a review. Curr Zool. 57:514–530.

[CIT0069] Soma M , Hiraiwa-Hasegawa M, Okanoya K. 2009. Early ontogenetic effects on song quality in the Bengalese finch *Lonchura striata* var. *domestica*: laying order, sibling competition, and song syntax. Behav Ecol Sociobiol. 63:363–370.

[CIT0070] Soma M , Takahasi M, Ikebuchi M, Yamada H, Suzuki M, Hasegawa T, Okanoya K. 2006. Early rearing conditions affect the development of body size and song in Bengalese finches. Ethology. 112:1071–1078.

[CIT0071] Spencer KA , Buchanan KL, Goldsmith AR, Catchpole CK. 2003. Song as an honest signal of developmental stress in the zebra finch (*Taeniopygia guttata*). Horm Behav. 44:132–139. 13129485 10.1016/s0018-506x(03)00124-7

[CIT0072] Spencer KA , Wimpenny JH, Buchanan KL, Lovell PG, Goldsmith AR, Catchpole CK. 2005. Developmental stress affects the attractiveness of male song and female choice in the zebra finch (*Taeniopygia guttata*). Behav Ecol Sociobiol. 58:423–428.

[CIT0073] Tchernichovski O , Nottebohm F. 1998. Social inhibition of song imitation among sibling male zebra finches. Proc Natl Acad Sci USA. 95:8951–8956. 9671785 10.1073/pnas.95.15.8951 PMC21183

[CIT0074] Therneau T. 2012. coxme: mixed effects Cox models (R package version 2.2-18.1). Vienna, Austria: R Foundation for Statistical Computing.

[CIT0075] Therneau T. 2023. A package for survival analysis in R (R package version 3.5-0). https://CRAN.R-project.org/package=survival [Accessed on 2023 Jan 20].

[CIT0076] Therneau T , Grambsch P. 2000. Modeling survival data: extending the Cox model. New York: Springer. ISBN 0-387-98784-3.

[CIT0077] Ton R , Hurley LL, Griffith SC. 2021. Higher experimental ambient temperature decreases female incubation attentiveness in Zebra Finches (*Taeniopygia guttata*) and lower effort yields negligible energy savings. Ibis. 163:1045–1055.

[CIT0078] van der Waaij EH , van den Brand H, van Arendonk JAM, Kemp B. 2011. Effect of match or mismatch of maternal–offspring nutritional environment on the development of offspring in broiler chickens. Animal. 5:741–748. 22439996 10.1017/S1751731110002387

[CIT0079] Vleck CM. 1981. Energetic cost of incubation in the zebra finch. Condor. 83:229–237.

[CIT0080] Wardle GM , Pavey CR, Dickman CR. 2013. Greening of arid Australia: new insights from extreme years. Austral Ecol. 38:731–740.

[CIT0081] Weinstock M. 2008. The long-term behavioural consequences of prenatal stress. Neurosci Biobehav Rev. 32:1073–1086. 18423592 10.1016/j.neubiorev.2008.03.002

[CIT0082] Wickham H. 2016. ggplot2: elegant graphics for data analysis. New York: Springer-Verlag. ISBN 978-3-319-24277-4. https://ggplot2.tidyverse.org [Accessed on 2023 Jan 9].

[CIT0083] Zanette L , Clinchy M, Sung H-C. 2009. Food-supplementing parents reduces their sons’ song repertoire size. Proc R Soc B Biol Sci. 276:2855–2860. 10.1098/rspb.2009.0450 PMC2839954 19457889

[CIT0084] Zann RA. 1996. The Zebra Finch: a synthesis of field and laboratory studies. Oxford: Oxford University Press.

[CIT0085] Zann R , Cash E. 2008. Developmental stress impairs song complexity but not learning accuracy in non-domesticated zebra finches (*Taeniopygia guttata*). Behav Ecol Sociobiol. 62:391–400.

